# Adrenal tumors in patients with neuroendocrine neoplasms

**DOI:** 10.1007/s12020-024-03810-7

**Published:** 2024-04-06

**Authors:** Henrik Falhammar, Adam Stenman, C. Christofer Juhlin, Anna Kistner

**Affiliations:** 1 Department of Endocrinology, Karolinska University Hospital, 171 77 Stockholm, Sweden; 2 Department of Molecular Medicine and Surgery, Karolinska Institutet, 171 76 Stockholm, Sweden; 3 Department of Breast, Endocrine Tumors and Sarcoma, Karolinska University Hospital, 171 76 Stockholm, Sweden; 4 Department of Oncology-Pathology, Karolinska Institutet, 171 76 Stockholm, Sweden; 5 Department of Pathology and Cancer Diagnostics, Karolinska University Hospital, 171 77 Stockholm, Sweden; 6 Department of Nuclear Medicine, Karolinska University Hospital, Stockholm, Sweden

**Keywords:** Neuroendocrine tumor, Neuroendocrine cancer, Adrenalectomy, Adrenal metastasis, Adrenal tumor, 68Gallium-DOTATOC-PET/CT

## Abstract

**Purpose:**

To study the prevalence of primary adrenal tumors and adrenal metastases in patients with neuroendocrine neoplasms (NENs) and describe these in detail. NENs can be further divided into neuroendocrine tumor (NET) and neuroendocrine carcinoma (NEC).

**Methods:**

A review of medical files was conducted for all patients who underwent a ^68^Gallium-DOTATOC-PET/CT during 2010−2023 or adrenalectomy during 1999-2023 at the Karolinska University Hospital.

**Results:**

In total, ^68^Gallium-DOTATOC-PET/CT was performed on 1750 individuals with NEN, among whom 12 (0.69%) had adrenal tumors. Of these, 9 (0.51%) were NEN metastases. Out of 1072 adrenalectomies, 4 (0.37%) showed evidence of NEN metastases. Thus, 16 patients with NEN exhibited adrenal tumors. The adrenal tumors were found on average 5 years after the NEN diagnosis and 19% of the adrenal tumors with simultaneous NEN were benign. Few had all adrenal hormones measured. None had an adrenal insufficiency nor an adrenal biopsy. Another synchronous metastasis was found in 69% at the time of the adrenal tumor discovery. During the median 2-year follow-up, 38% of the subjects had deceased (with the exclusion of individuals presenting supposedly benign adrenal tumors 31%) all due to tumor complications. A comparison between individuals identified through ^68^Gallium-DOTATOC-PET/CT and those who underwent adrenalectomy revealed a higher prevalence of NETs in the former group and NECs in the latter group.

**Conclusion:**

Adrenal primary tumors and adrenal metastases are infrequent occurrences in patients with NEN. Most cases involved the presence of NEN metastasis upon the initial discovery of adrenal tumors. The overall prognosis was found to be favorable.

## Introduction

Neuroendocrine neoplasms (NENs) are rare malignancies that can be divided into neuroendocrine tumors (NETs) with more indolent biological behavior and neuroendocrine cancers (NECs) with more aggressive biological behavior. According to the 2019 WHO classification, this division is mainly based on the Ki-67 proliferation index where NETs with well-differentiated histology can be further divided into grades G1 (Ki-67 < 3%), G2 (Ki-67 3–20%) and G3 (Ki-67 > 20%), while NECs exhibit high-grade histological features and a Ki-67 > 20%. Around 40-50% of all NENs are metastasized at initial diagnosis with liver and lymph node metastases being the most common [1]. In contrast to other malignancies, only occasional cases of adrenal metastasis from NEN have been reported [2–5]. The long-term prognosis of adrenal metastases from other cancers is unfavorable [6], but the outcomes in NEN is unclear, especially since NETs may have a very indolent course. Moreover, adrenal tumors are common in the general population, with the vast majority being adrenal cortical adenomas [6]. A proper workup for an adrenal tumor with hormone and imaging evaluation also needs to be done in patients with NEN. Benign adrenal tumors have been reported in one study to be more common in patients with NEN than in the general population [3].

The aim of the present study was to assess the prevalence of primary adrenal tumors and adrenal metastases in patients with NEN who underwent investigation using ^68^Gallium-DOTATOC-PET/CT or in those who underwent adrenalectomy. Moreover, the study also aims to describe the cases and compare the two groups.

## Methods

The imaging reports from all patients that had had a ^68^Gallium-DOTATOC-PET/CT performed at the Karolinska University Hospital since its introduction in January 2010 to October 2023 were reviewed. During the study period ^68^Gallium-DOTATOC-PET/CT was performed mainly in patients with NEN at diagnosis, 6 months after surgery with curative intention, during progression to evaluate treatment options such as additional surgery or ^177^Lutetium peptide receptor radionuclide therapy and before discharging the assumed cured patient. From our entire ^68^Gallium-DOTATOC-PET/CT cohort (2773 patients) 37% were not patients with NENs. Of the remaining cohort (1750 patients) 39% consisted of patients with ileal-NEN, 29% were NENs of pancreatic origin and 13% were lung carcinoids. The remaining 19% of the NENs had other primary tumor location. Moreover, by using the Systematized Nomenclature of Medicine (SNOMED) codes T93 (adrenal) and M82466 (metastasis malignant neuroendocrine tumor) histopathologically diagnosed adrenal NEN metastases from January 1999 (when the electronic search system using SNOMED codes was introduced) until December 2023 were found. Adrenalectomies were mainly performed in patients with functional adrenal tumors, suspected primary adrenal cancers or adrenal metastases, almost all with curative intent. The most frequent histopathological diagnosis post-adrenalectomy was adrenal cortical adenoma (32%), followed by 23% annotated as adrenal cortical hyperplasia, and 19% pheochromocytoma. The remainder of the cohort (26%) included neuroblastoma, adrenal cysts, adrenal cortical carcinoma, metastatic tumors (4.8%), as well as rare diagnoses such as myelolipoma, schwannoma and so on.

The medical records of the patients were subsequently examined for additional information. Simultaneously, the national population registry was scrutinized to determine whether the patient had passed away and, if so, the date of death.

The study was approved by the Swedish Ethical Review Authority and due to the retrospective nature of the study informed consent was waived.

### Statistical analysis

Non-parametric statistical analysis was used. Continuous data were analyzed using Kruskal−Wallis test while categorical data were analyzed using the Fischer exact test or if not possible Chi2 test. A *p* < 0.05 was considered statistically significant.

## Results

**Fig. 1:**
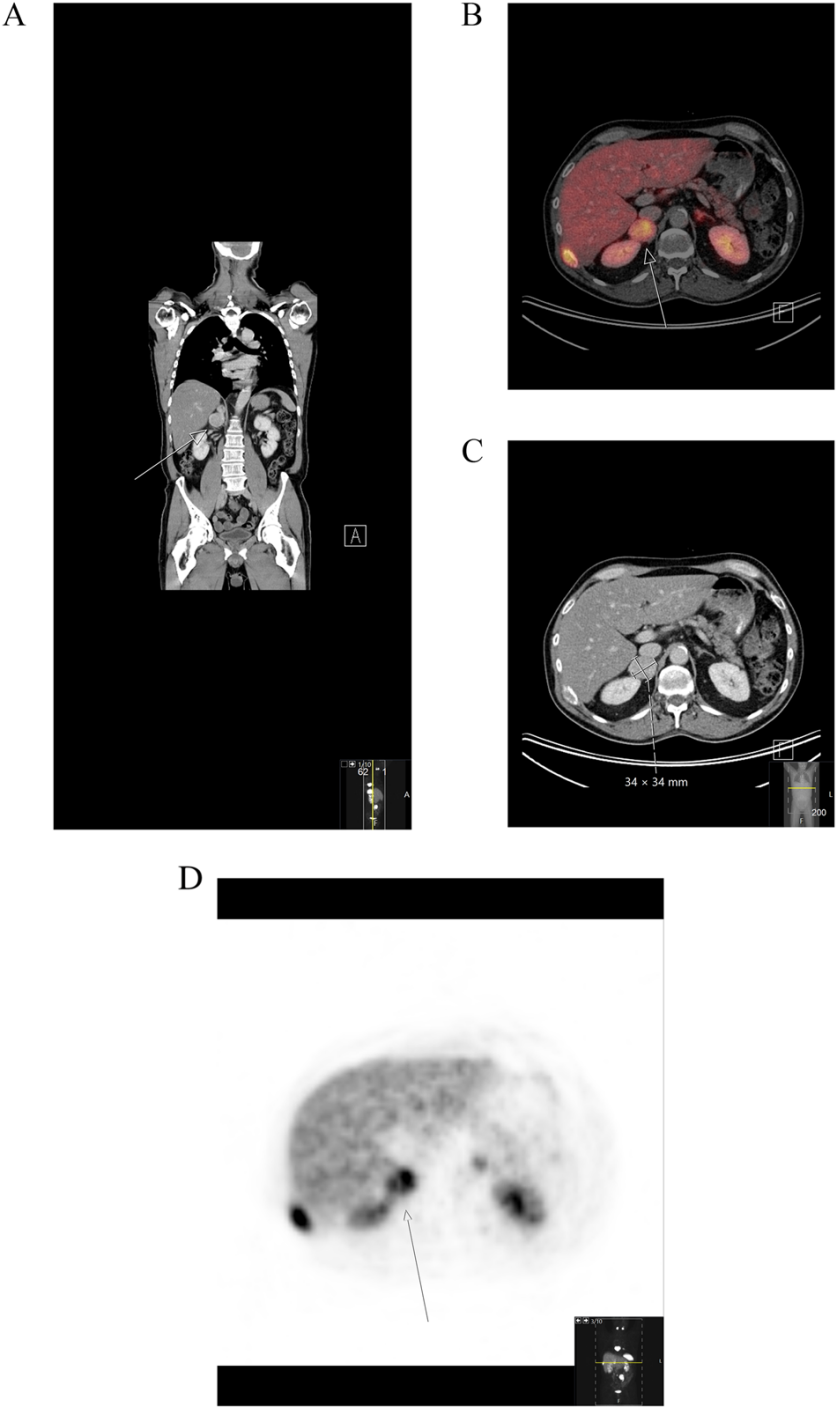
A right adrenal mass at ^68^Gallium-DOTATOC-PET/CT in a middle-aged male. CT image coronar projection (left **A**), fused CT (middle **B**), CT image (**C**) and maximum intensity projection (MIP) projection (**D** left) of the corresponding axial projection over the adrenal glands. The right adrenal (white arrow) is enlarged and demonstrates a diffuse distinct DOTATOC uptake, not seen in the left, normal-sized adrenal

**Fig. 2:**
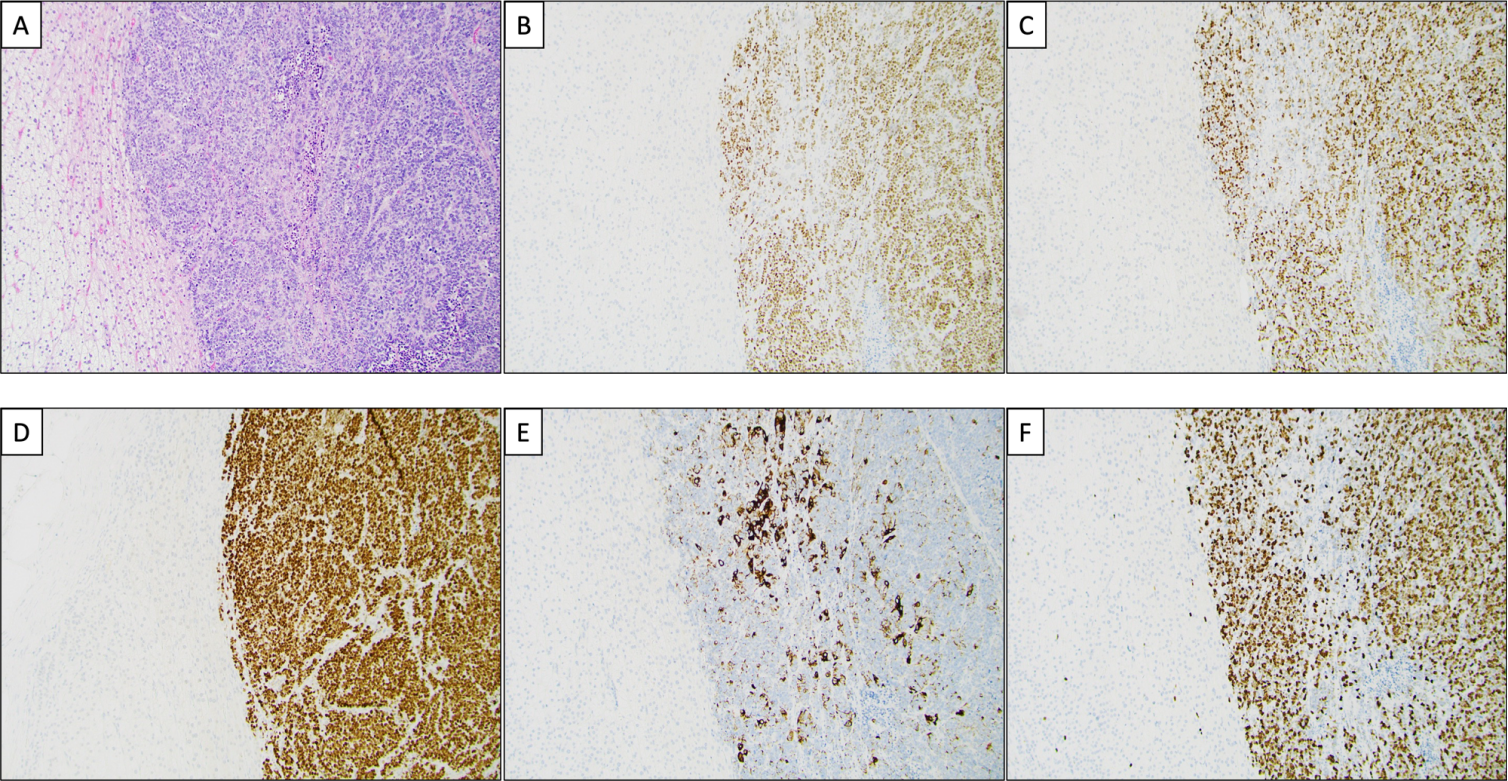
Histological and immunohistochemical features of a neuroendocrine carcinoma metastasis to the adrenal gland. **A** Routine hematoxylin and eosin staining depicting the normal adrenal cortex (left) and a metastatic neuroendocrine carcinoma (NEC) (right). The tumor is arranged in ribbons and solid sheets, with numerous mitotic figures and focal tumor necrosis. **B** The second-generation neuroendocrine marker ISLET1 is diffusely positive in tumor cells, as was first-generation marker synaptophysin (not shown). **C** INSM1 is also clearly positive, thus verifying the neuroendocrine nature of this lesion. **D** Diffuse TTF1 expression in the tumor cells. **E** CK7 is focally expressed within the tumor. The TTF1 and CK7 stains favored a pulmonary origin. **F** The Ki-67 labeling index is 80%

**Fig. 3:**
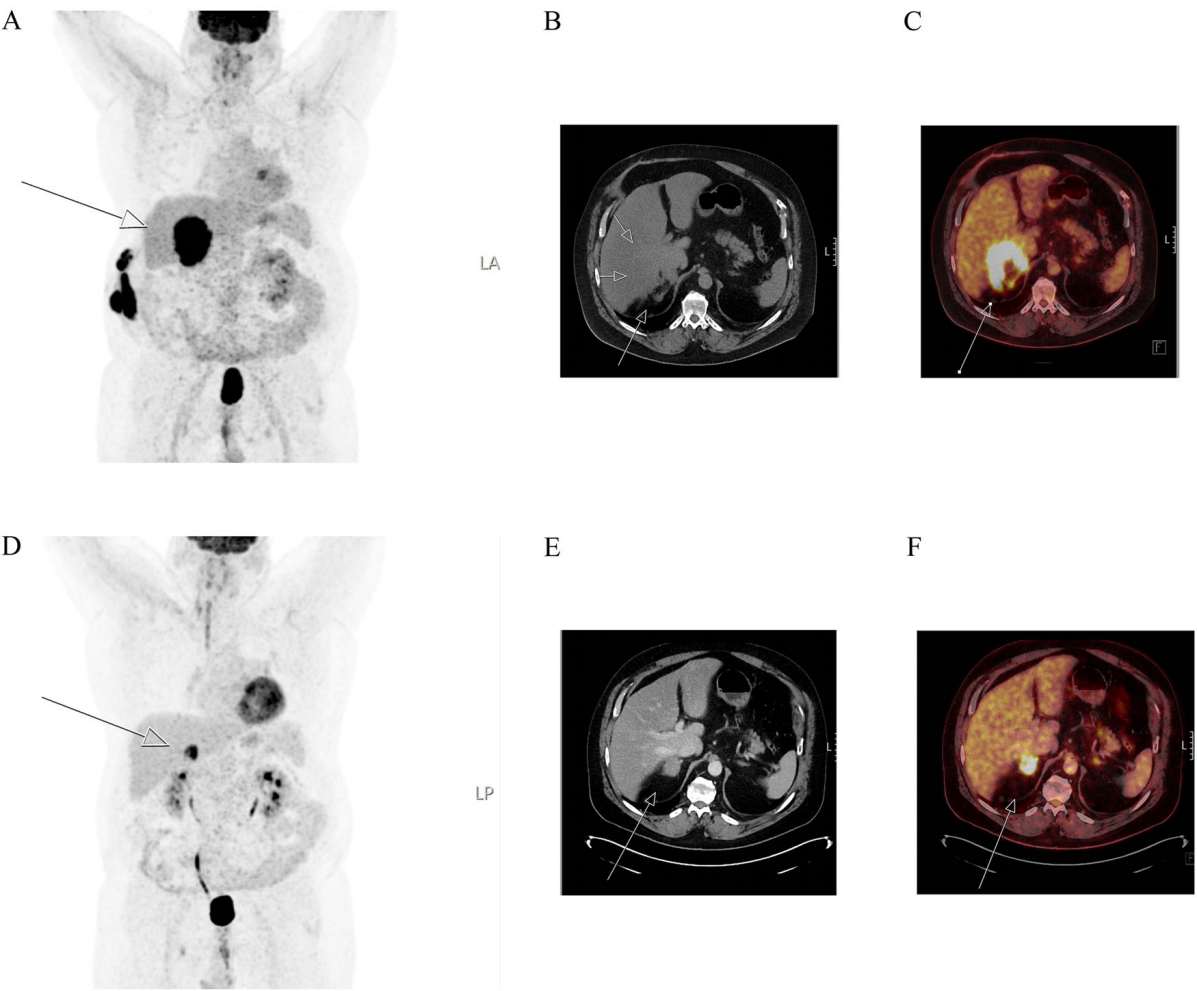
^18^F-FDG-PET/CT imaging of the same patient histologically and immunohistochemically demonstrated in Fig. 2. Maximum intensity projection (MIP) image (left **A**, and **D**), CT images (middle **B** and **E**) and fused images (right **C** and **F**) of ^18^F-FDG-PET/CT show the adrenal gland metastasis (white arrow) in the right adrenal. The metastasis increased in size from 4 (**E**, **F**) to 9 (**A**−**C**) centimeters between examinations, which were carried out at an 8-month interval

In total, 4543 ^68^Gallium-DOTATOC-PET/CT investigations were performed in 2773 individuals. Of these, 1750 were of NEN origin; and from this cohort 12 (0.69%) had an adrenal tumor of which 9 (0.51%) were considered to be metastases from NENs (Fig. 1). From 1072 adrenals that were available for histological work-up, 4 (0.37%) showed a metastasis from a NEN (Figs. 2 and 3). Thus, 16 patients with NEN and an adrenal tumor were found. Details on these patients can be found in Table 1. The median age of NEN diagnosis was 59.5 years and twice as many were males compared to females. Half were diagnosed with ileal-NET and about a fifth with NEC. There was a wide span in terms of the proliferation rate (as measured by Ki-67). The adrenal tumors were found around 5 years after the NEN diagnosis and in the vast majority the adrenal lesion was seen on the first PET/CT or scintigraphy performed. Most adrenal masses were left sided and only a fifth were lipid rich indicating a benign adrenal tumor. Approximately half of the patients had at least one adrenal hormone measured, but only a quarter had all three adrenal hormones measured, as recommended when identifying an adrenal tumor. None of the patients had primary adrenal insufficiency (PAI) or underwent an adrenal biopsy. At the time of adrenal tumor discovery, approximately two-thirds of the patients had another metastasis, most commonly in the liver. During the median follow-up of 2 years, 38% of the patients had died, all due to tumor complications. If only considering those patients with adrenal metastases 31% were dead within 2 years. One of these deaths was attributed to suspected ectopic Cushing syndrome from the ileal-NET.

**Table 1:** Characteristics and outcomes of patients with neuroendocrine neoplasms and adrenal masses, also divided into two groups which are compared

	All patients with NEN and adrenal masses (*n* = 16)	Patients with NEN and ^68^Ga-DOTATOC PET/CT (*n* = 12)	Patients with NEN and adrenalectomy (*n* = 4)	*P* value
Age at NEN diagnosis (years)	59.5 (39−80)	62 (39−80)	58.5 (52−75)	0.99
Sex	F *n* = 5, M = 11	F *n* = 5, M *n* = 7	F *n* = 0, M = 4	0.24
Primary tumor				0.038
Ileal-NET (*n*)	8 (50%)	8 (67%)		
Pancreatic-NET (*n*)	2 (13%)	2 (17%)		
Atypical bronchial-NET (*n*)	2 (13%)	1 (8%)	1 (25%)	
Small cell abdominal-NEC (*n*)	1 (6%)	1 (8%)		
Skin-NEC^a^ (*n*)	1 (6%)		1 (25%)	
Gastric-NET (*n*)	1 (6%)		1 (25%)	
Pulmonary large cell-NEC (*n*)	1 (6%)		1 (25%)	
Ki-67	7.2% (0.1−80%)	1.9% (0.1−80%)	47.5% (4.5−80%)	0.091
Grade	2 (1−3)	1 (1−3)	2.5 (2−3)	0.077
Stage	3 (2−4)	4 (2−4)	3 (2−3)	0.062
When adrenal mass seen on PET/CT or scintigraphy				
Age at diagnosis of adrenal tumor (years)	64.5 (39−80)	66.5 (39−80)	60.5 (54−78)	0.96
First PET/CT or scintigraphy the adrenal mass was seen (*n*)	14 (88%)	10 (83%)	4 (100%)	1
Adrenal mass known before (*n*)	7 (44%)	3 (25%)	4 (100%)	<0.001
Adrenal mass strongly positive on PET/CT or scintigraphy^b^	13 (81%)	9 (75%) (^68^Ga-DOTATOC)	4 (100%) (3 ^18^F-FDG, 1 ^111^In-pentetreotide)	0.52
Bilateral	1 (6%)	1 (8%)	0 (0%)	1
Left *n*, size (mm)	11 (69%), 18 (10−80)	9 (75%), 15 (10−80)	2 (50%), 35.5 (28−43)	0.55, 0.45
Right *n*, size (mm)	6(38%), 70 (34−90)	4 (33%), 70 (36−85)	2 (50%), 62 (34−90)	0.60,0.90
Total size (mm)	26.5 (10−160)	19 (10−160)	38.5 (28−90)	0.68
Lipid rich tumor	3 (19%)	3 (25%)	0 (0%)	0.53
Considered to be NEN metastases^c^	13 (81%)	9 (75%)	4 (100%)	0.52
Adrenal hormones assessed^d^				
Any (*n*)	9 (56%)	5 (42%)	4 (100%)	0.089
All (*n*)	4 (25%)	2 (17%)	2 (50%)	0.24
Other metastasis at the time of adrenal mass discovery (*n*)	11 (69%)	9 (75%)	2 (50%)	0.55
Liver metastases (*n*)	8 (50%)	8 (67%)		
Abdominal lymph node metastases (*n*)	7 (44%)	7 (58%)		
Pancreatic metastases (*n*)	1 (6%)	1 (8%)		
Peritoneal carcinosis (*n*)	1 (6%)	1 (8%)		
Bone metastases (*n*)	3 (19%)	2 (17%)	1 (25%)	
Lung metastases (*n*)	4 (25%)	2 (17%)	2 (50%)	
Neck lymph node metastases (*n*)	1 (6%)	1 (8%)		
Brain metastases (*n*)	1 (6%)	1 (8%)		
No metastasis	5 (31%)	3 (25%)^e^	2 (50%)^f^	0.55
Follow-up since adrenal mass diagnosis	2 years (0−11 years)	1 year (0−11 years)	3 years (2−9 years)	0.25
Dead (*n*)	6 (38%)	3 (25%)	3 (75%)	0.11
Cause of death (*n*)	4 (25%) metastases 1 (6%) carcinoid crisis 1 (6%) Cushing syndrome	1 (33%) carcinoid crisis 1 (33%) metastasis 1 (33%) Cushing syndrome	3 (100%) metastases	

^a^Merkel Cell Carcinoma

^b^All primary tumors were strongly positive as well

^c^Cortisol, metaneprhines, aldosterone and renin concentrations, in the any group at least the metanephrines were controlled. One patient with ileal NET develops Cushing syndrome suspected to be ectopic

^d^In those not having histology this assumption was built on a combination of radiological parameters, e.g., a strong uptake on PET/CT or scintigraphy, lipid contents, growth pattern and other radiological features

^e^1 patient developed extensive liver metastases within 2 years. NEN, neuroendocrine neoplasia. NET, neuroendocrine tumor. NEC, neuroendocrine carcinoma

^f^1 patient developed a brain metastasis within 3 years

When comparing those identified on the ^68^Gallium-DOTATOC-PET/CT with those who underwent adrenalectomy, the site of the primary tumor differed, with more being ileal-NETs in the former group and NECs in the latter group. There were tendencies that those who underwent adrenalectomies had higher Ki-67 and tumor grades but lower tumor stages and median age at the discovery of the adrenal tumor. All patients with an adrenalectomy had the adrenal tumor known prior to the PET/CT or scintigraphy. All other parameters were similar between the groups (Table 1).

## Discussion

This is the largest study to date describing NEN metastases to the adrenals, which were rare, occurring in only 0.51% of all patients with NEN who underwent a ^68^Gallium-DOTATOC PET/CT, slightly higher compared with the prevalence (0.37%) in patients undergoing adrenalectomy. In those with ^68^Gallium-DOTATOC-PET/CT all but one had a NET while in those with adrenalectomy it was the opposite, i.e., all but one had a NEC. The adrenal tumors were diagnosed around 5 years after the NEN diagnosis. In most patients with ^68^Gallium-DOTATOC-PET/CT the adrenal tumor was a metastasis while in all having an adrenalectomy the tumor was a metastasis by definition. Only around half of the patients had at least one adrenal hormone measured. The majority had NEN metastases at the time of adrenal tumor discovery. During the follow-up of around 2 years 38% had died, all to tumor related. The differences between the two groups were otherwise small.

Adrenal tumors are common, affecting around 2% of the general population but increasing with age [7]. Most are benign and nonfunctioning and not considered to affect health [7], even if this has been questioned recently [8]. In contrast, adrenal metastases have poor prognosis with almost all of those affected being dead within 2 years [6, 9]. However, in our cohort only 31% with adrenal metastases were dead within 2 years. While NEN accounts for only 1% of all adrenal metastases the majority originates from the lung (39%), genitourinary (27.6%) and gastrointestinal organ systems (13.6%) [9]. Moreover, around 25% will have bilateral adrenal metastases [9], compared to only 6% in our cohort. PAI can occur in those with bilateral adrenal metastases and then affect around 3−12.4% [9, 10]. These differences probably account to the more indolent nature of NETs while most of our NECs, which generally have a poor prognosis, had an adrenalectomy. An adrenalectomy is mainly done if you believe you can improve the prognosis [11]. Even though we did not find PAI in our case with bilateral adrenal metastases, no hormonal evaluation was done. Since some symptoms of PAI are similar to malignancy (fatigue, pain, nausea) [12], we cannot totally exclude that a PAI was present.

Hormonal evaluation was done in only around half of our cohort, but then only a quarter had all adrenal hormones (serum/plasma cortisol post-dexamethasone, metanephrines and aldosterone/renin ratio if hypertension or hypokalemia are present) done which are recommended when evaluating adrenal tumors [7]. This is despite many of patients with NENs being followed-up by endocrinologists in our center. In the study by Kanakis et al. only 7.7% had hormonal evaluation at all and in the study by Mao et al. evaluating adrenal metastases in general only 31.8% had had catecholamines performed and 30.2% had an evaluation by an endocrinologist [9]. Excluding a pheochromocytoma is important before surgery or biopsy to avoid a hypertensive crisis which can be lethal [13], and all our patients with an adrenalectomy had had at least the catecholamines measured. Interestingly, one of the patients with ileal-NET developed ectopic Cushing’s syndrome which also was the cause of death in this patient. Approximately 1% of gastrointestinal-pancreatic-NET develop ectopic Cushing’s syndrome of which pancreatic-NET being the most common cause [3, 14].

Adrenal tumor size in our patients with NENs was similar to other adrenal metastases which are usually 30 mm [9]. Interestingly, around two thirds of our adrenal masses were left-sided which is in accordance with many studies of adrenal tumors [7]. Left-sided adrenal masses may be more easily detected on imaging. This is supported by an imaging study were left-sided adrenal tumors were more common in those with a tumor size <30 mm while in larger there was no difference [15]. This can also be suggested by our cohort where those who had had adrenalectomy had no imbalance of side location of the masses since the tumor sizes were around 30 mm or larger in these cases. Thus, smaller adrenal tumors in patients with NENs are probably missed during radiological evaluation. This is confirmed by the fact that very few benign adrenal tumors were found in the present study in spite of most patients being in an age where these are common. Also, bilateral disease may be underestimated by the same reasoning. Moreover, it is highly probable that the radiologists, reporting on the imaging, focused on identifying metastases and omitted mentioning potentially benign adrenal tumors, as these were unlikely to impact the management of the patients. This may explain the difference between our study and the study by Kanakis et al. where all abdominal imaging of 383 patients with NETs were reviewed and a prevalence of 8.4% of an adrenal tumor (22% bilateral) was found but only one had a suspected adrenal NEN metastases (0.26%) [3]. The prevalence of adrenal NEN metastases were slightly lower than our 0.51% and we would probably find more benign adrenal adenomas if we reviewed all images instead of only reviewing the radiological reports.

Surgical removal of metastases have historically not been performed often [11]. However, modern systemic cancer treatment has led to increased survival with many living with the cancer or experiencing recurrence. Surgical removal of metastases has enhanced survival in many cancers [11], including radical liver metastases resection in NENs [16]. There is an increased survival in patients having their adrenal metastasis being removed [9], but only occasional case reports exist where adrenalectomy in patients with NENs have been described [2, 4]. In the present study we add to the literature another four patients with NENs having adrenalectomy for their adrenal metastases with all having reasonable outcomes with 2−9 years survival (median 3 years) after the procedure. It should be noted that three of these patients had a NEC and NEC are known to have an extremely poor prognosis [1].

### Limitations

The prevalence numbers are only estimations because we did not have detailed characteristics of the two cohorts from which we included patients with NENs and adrenal tumors. Ideally, the study should provide the tumor stage at diagnosis, clinical course, reasons for performing the ^68^Gallium-DOTATOC-PET/CT, and the presence of any genetic syndromes. However, the study’s primary aim was solely to provide an estimate of adrenal NEN metastases prevalence, instead concentrating on describing the identified cases in detail. It should be noted that ^68^Gallium-DOTATOC-PET/CT is not a suitable modality in NEC, where most tumors are not visualized due to high proliferation, and as such, some cases of NECs may have been missed. Even though this is the largest study to date on patients with NENs metastatic to the adrenal, the number of participants is still limited, preventing us from conducting subgroup analyses of the different tumor types. Moreover, it is a retrospective study with its inherited limitations such as missing data and the potential of information bias. Furthermore, we solely reviewed imaging reports; consequently, there is a possibility that we may have overlooked some adrenal tumors that could have been identified if we had examined the actual images. Lastly, as this is a single-center study, the results may not be generalizable to a broader population.

## Conclusion

Adrenal metastases due to NENs occurred in only 0.51% of all patients with NEN who had undergone a ^68^Gallium-DOTATOC-PET/CT and a mere 0.37% of all patients who had undergone adrenalectomy. Adrenalectomy was predominantly performed in patients diagnosed with NEC, with roughly half of the patients having at least one adrenal hormone measured. The majority of cases exhibited NEN metastases at the time of adrenal tumor discovery. The overall prognosis was reasonable. Larger-scale multicenter studies are desirable in the future for investigating this rare condition.

## Data Availability

Restrictions apply to the availability of data generated or analyzed during this study to preserve patient confidentiality or because they were used under license.
